# Supplementary material for: Markers of acute toxicity of DDT exposure in pancreatic beta-cells determined by a proteomic approach
Source: PLoS One. 2020 Oct 26;15(10):e0229430. doi: 10.1371/journal.pone.0229430 (PMC7588079; doi:10.1371/journal.pone.0229430)

Figure S1: Cell viability of NES2Y exposed to 150 µM DDT for 72 hours

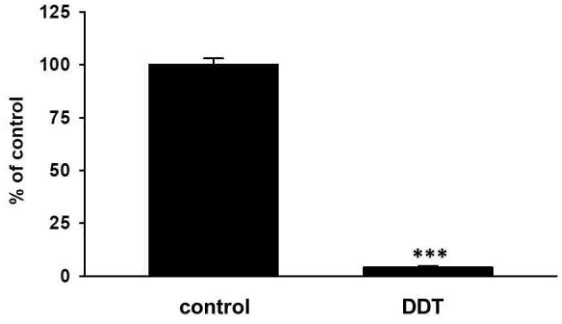

Viability of NES2Y cells exposed to 150 µM DDT for 72 hours determined by neutral red assay. We used cells exposed to DMSO (final concentration of 0.5%) as solvent control. The graph represents a mean of four independent experiments. *** means statistically significant difference ($p < 0.001$) when compared to the solvent control (student's t-test).

Western blot results.

For our western blots, we dyed the membrane with blotted proteins using Ponceau, then cut the membrane into strips and incubated them with chosen primary antibodies. After taking pictures, we stripped the membranes and used them for the detection of another protein. We used NES2Y cells exposed to stearic acid for 24 hours as a positive control; nevertheless, in some cases, the positive control showed a much higher expression of the tested protein than the samples, so we had to cover the SA bend to get satisfying results of our samples.

Figure S2. Western blot results of CHOP – uncropped membrane

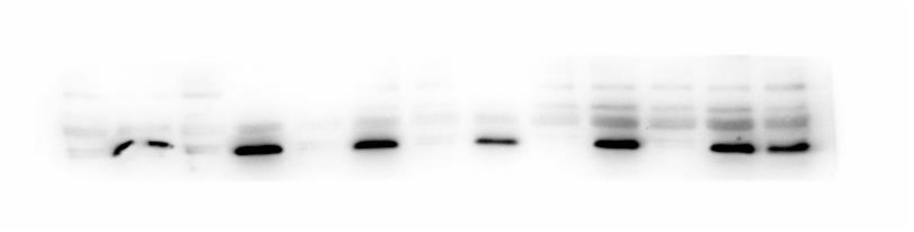

Figure S3. Western blot results of GRP78 – uncropped membrane

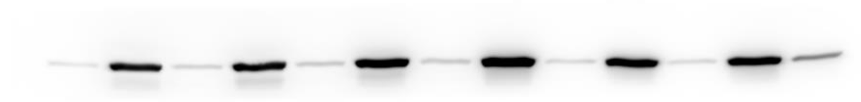

Figure S4. Western blot results of cleaved caspase-6 – uncropped membrane

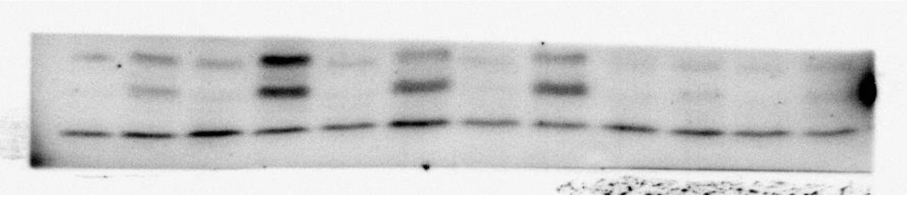

Figure S5. Western blot results of cleaved caspase-7 – uncropped membrane

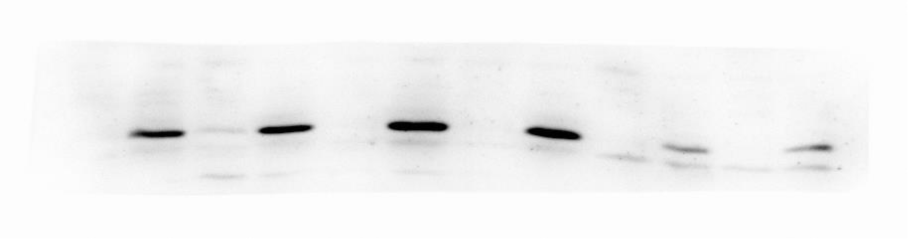

Figure S6. Western blot results of cleaved caspase-8 – uncropped membrane

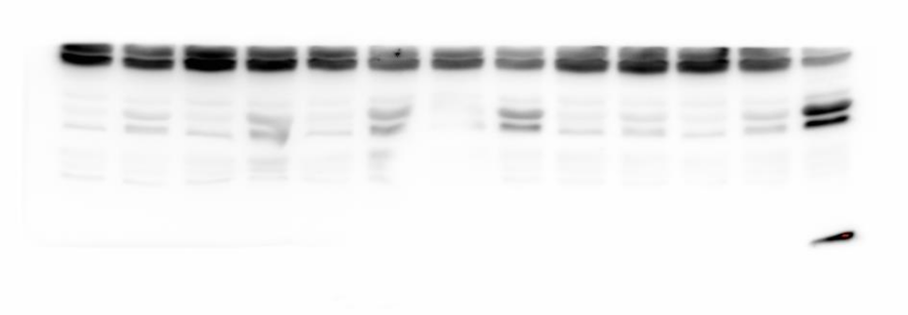

Figure S7. Western blot results of cleaved caspase-9 – uncropped membrane

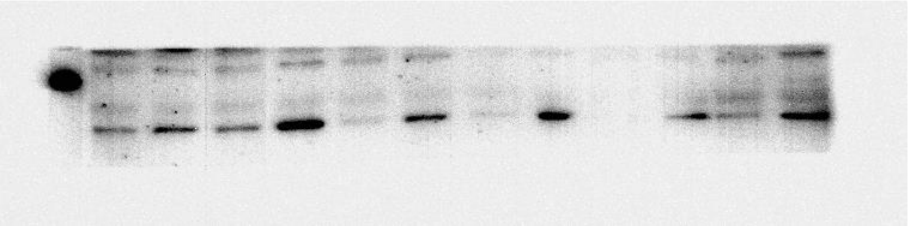

Figure S8. Western blot results of PARP – uncropped membrane PARP

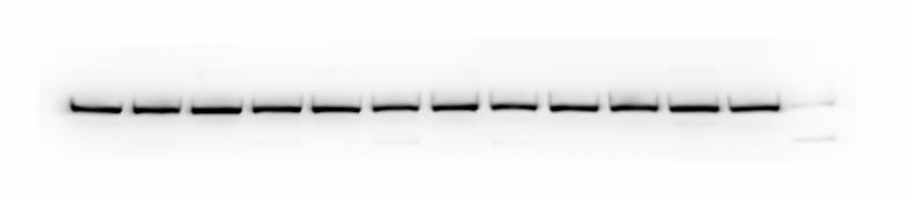

Figure S9. Western blot results of cleaved PARP – uncropped membrane

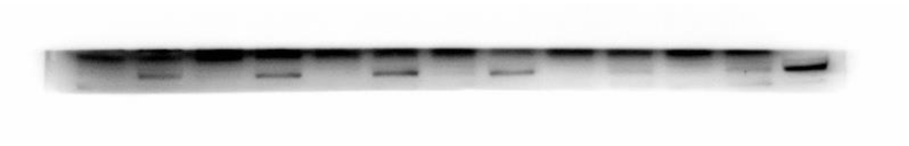

Figure S10. Western blot results of actin – uncropped membrane

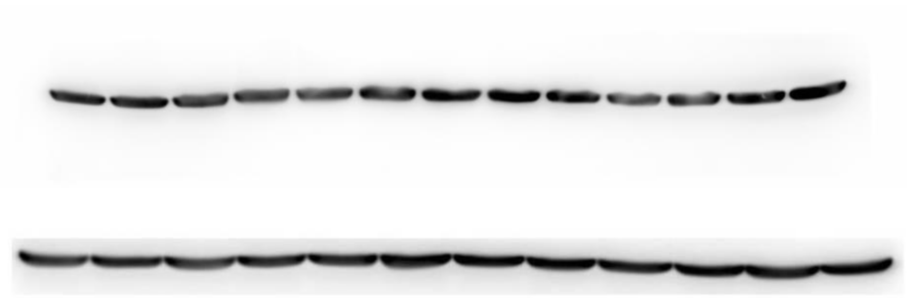

Figure S11: 2-D electrophoresis results after 24-hour exposure

Control **DDT**

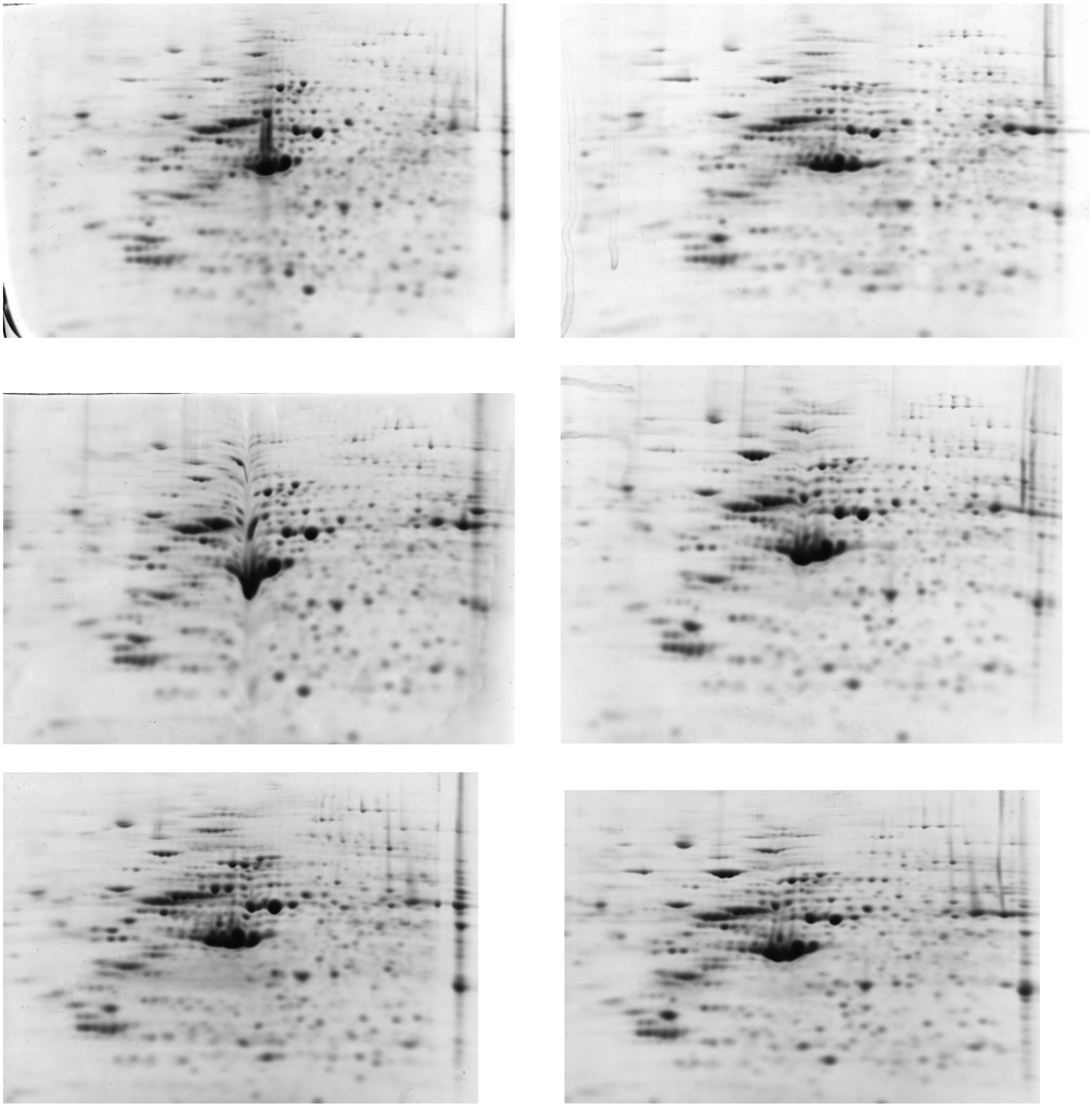

Figure S12: 2-D electrophoresis results after 30-hour exposure

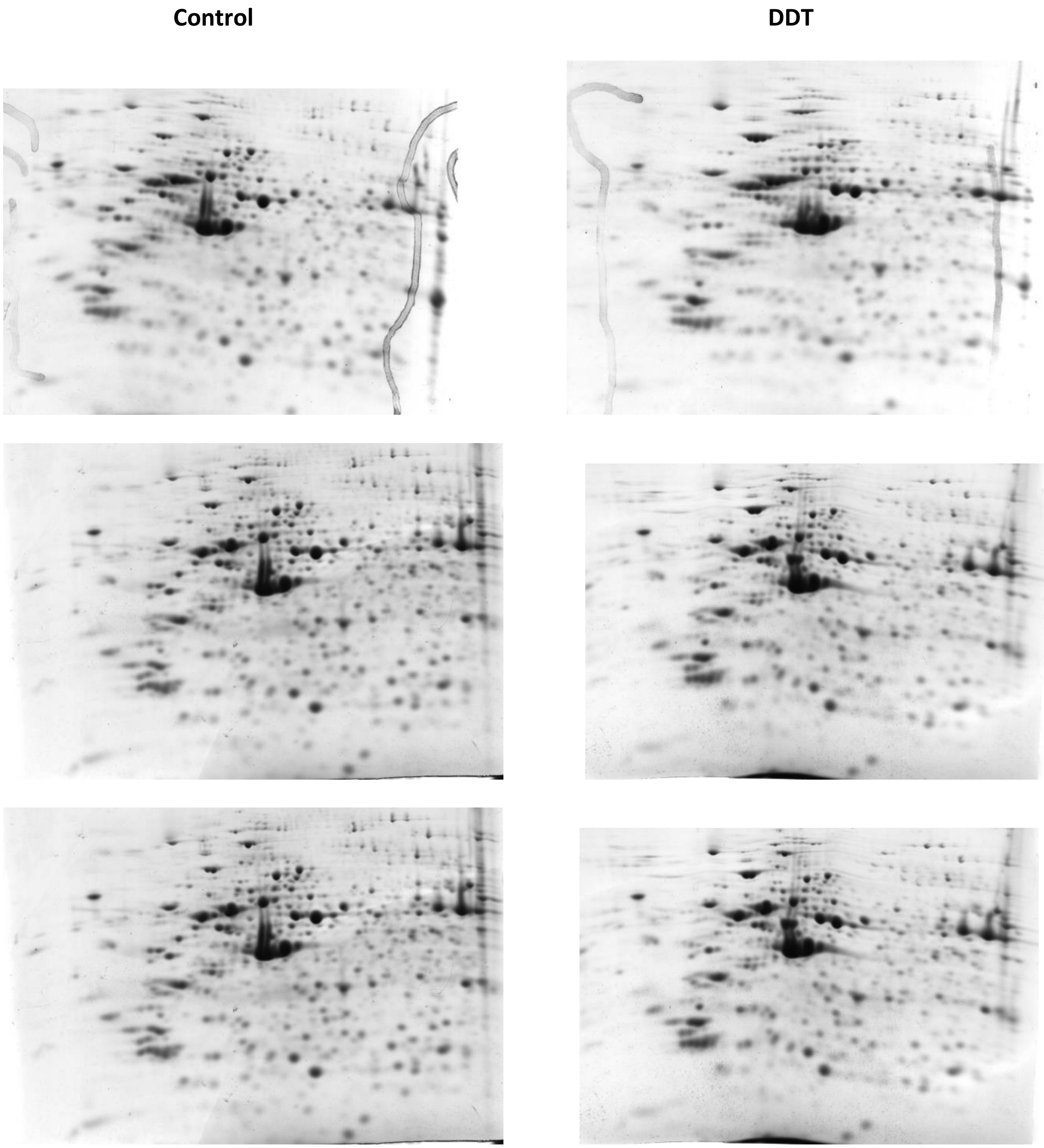

Supplement: S1 File — (PDF) [file pone.0229430.s001.pdf]
